# Alveolar bone exostoses following orthodontic treatment. Diagnostic considerations and clinical management

**DOI:** 10.1002/ccr3.7000

**Published:** 2023-03-07

**Authors:** Adith Venugopal, Noem Bunthouen, Hasan Sabah Hasan, Krenare Agani, Andrea Butera, Nikhilesh R. Vaid

**Affiliations:** ^1^ Department of Orthodontics University of Puthisastra Phnom Penh Cambodia; ^2^ Department of Orthodontics, Saveetha Dental College and Hospitals, Saveetha Institute of Medical and Technical Sciences Saveetha University Chennai India; ^3^ Private Practice Phnom Penh Cambodia; ^4^ Orthodontic Department, Khanzad Teaching Center General Directorate of Hawler‐Ministry of Health Erbil Iraq; ^5^ Private Practice Bundesland Germany; ^6^ Unit of Dental Hygiene, Section of Dentistry, Department of Clinical, Surgical, Diagnostic and Pediatric Sciences University of Pavia Pavia Italy

**Keywords:** alveolar buccal exostoses, bone exostoses, buttressing bone formation, palatal exostosis, torus Mandibularis, torus Palatinus

## Abstract

Alveolar bone exostoses (ABE) are benign localized convex outgrowths of buccal or lingual bone, which could be delineated from the surrounding cortical plate, also known as a buttress bone formation. Our review and case series demonstrate the development of alveolar bone exostoses during orthodontic therapy. It is crucial to keep in mind that every case presented had a history of palatal tori. In our clinical observations, higher precedence of ABE development was seen in participants during incisor retraction, especially with preexisting palatal tori. Additionally, we have successfully demonstrated surgical techniques to eliminate ABE in the event that self‐remission does not occur once orthodontic forces are discontinued.

## Graphical abstract

Alveolar bone exostoses (ABE) are benign localized convex outgrowths of buccal or lingual bone from the cortical plate, often known as buttress bone development. Our review and case series shows ABE following orthodontic treatment. In case self‐remission fails after orthodontic forces are removed, we have shown surgical methods to correct ABE.

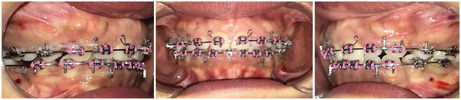

## 1 INTRODUCTION

An exostosis is a peripheral overgrowth of bone that is benign and has an undetermined cause. It may be an alveolar surface enlargement that is nodular, flat, or pedunculated. Torus palatinus (TP), torus mandibularis (TM), and alveolar bone exostoses (ABE) are the three anatomical terms for these lesions in the jaws, respectively.^1^

Occasionally the same person may develop multiple exostoses. In young, dentate subjects, they may manifest as discrete, isolated bony growths on the facial alveolar bone or, less frequently, as multiple exostoses in the maxilla (torus palatinus) and mandible (mandibular tori)^2^ (Table 1).

**TABLE 1:** Clinical classification.

Location	Type
Midline of the Palate	Torus palatinus (TP)
Bilaterally in the lingual surface of the mandible, above the mylohyoid line, most commonly seen in the canine and premolar areas	Torus mandibularis (TM)
Buccal aspect of the maxilla or mandible, usually in the premolar and molar areas.	Alveolar buccal exostosis
Palatal aspect of the maxilla, and the most common location is the tuberosity area	Palatal exostosis

Numerous authors have investigated the etiology of tori, but no consensus has emerged yet. Some of the speculated causes include genetics, environmental factors, masticatory hyperfunction, and continued growth.^3^, ^4^, ^5^, ^6^

Torus palatinus (TP) and torus mandibularis (TM) tori prevalence varies with sample population, ranging from 0.4% to 66.5% and 0.5% to 63.4%, respectively. Racial differences seem to be considerable with a significant prevalence among Asian and Eskimo communities.^7^, ^8^ There have also been reports of gender‐specific variations in tori prevalence. The majority of authors asserted that while TM presented more in men than women, TP mostly prevailed in women.^9^ Table 2 provides a summary of some of the theories and mechanisms that might be involved in their formation.

**TABLE 2:** Etiology, theories, and possible mechanisms.

Etiology	
Genetic factors	Eggen S et al.^4^ Reichart PA et al.^9^ Antoniades DZ et al.^10^ Regezi JA et al.^11^ Suzuki M et al.^12^ Gorsky M et al.^13^
Environmental factors	King DR et al.^14^ Haugen LK et al.^15^
Inter‐play of multifactorial genetic and environmental factors, quasi‐continuous genetic, or threshold theory	Antoniades DZ et al.^10^ Gorsky M et al.^13^ Neville BW et al.^16^ Seah YH et al.^17^
Nutrients: saltwater fish consumption	Eggen S et al.^18^
Masticatory hyperfunction	Reichart PA et al.^9^ King DR et al.^14^ Haugen LK et al.^15^ Eggen S et al.^19^ Kerdpon D et al.^20^ Matthews GP.^21^ Johnson OM.^22^
Continued growth	Topazian DS et al.^23^
Theories and possible mechanism	
Bone flexion	Horning GM et al.^6^
Internal functional stresses	Sennerby L et al.^24^
Periosteal bone or mechanical factor of ministrain	Marx RE, Garg AK.^25^
Periosteal trauma	Chambrone LA et al.^26^ Echeverria et al.^27^ Otero‐Cagide et al.^28^
Chronic irritation, Subpontine Osseous Hyperplasia (SOH)	Burkes et al.^29^ Brooks JK et al.^30^ Wasson DJ et al.^31^
Periosteal activation	Nikitakis et al.^32^
Vascular disruption as a consequence of the surgical trauma	Svindland et al.^33^

Alveolar bone exostoses (ABE), also known as buttress bone formations, are benign, isolated, convex outgrowths of the buccal or lingual bone that may be distinguished from the surrounding cortical plate.^6^

Glickman and Smulow^34^ distinguished between two categories of buccal alveolar bone enlargement: exostosis and lipping. Exostoses were described as harmless, isolated, convex outgrowths of the buccal or lingual bone that could be distinguished from the cortical plate around them. On the contrary, identifiable thickenings in the alveolar bone at the direct crestal edge were referred to as buccal lippings.^6^

Alveolar bone exostoses are multiple bony nodules that are found less often than tori. In contrast, Horning et al.^6^ found ABE or lipping to be present fairly frequently, with 76.9% of all the specimens having at least one, in a study on 52 skulls with complete dentition.

### 1.1 Clinical presentation and differential diagnosis

A clinical examination and radiographic evidence should be used to make the diagnosis. These exostoses must be distinguished from pathology originating from the bone (osteomyelitis, osteoma, and osteosarcoma) or the gingiva (enlargement of the gingiva). The absence of inflammation‐related symptoms allows one to rule out inflammatory gingival hypertrophy and osteomyelitis. Osteoma, osteosarcoma, and other intra‐bony pathologies may be identified based on radiographic and histological findings.^35^, ^36^

Buccal exostoses are bilateral, smooth bony growths that form on the facial aspect of the mandibular and/or maxillary alveolus. It often occurs in the premolar‐molar region.^37^ When palpated, the exostoses feel like a solid, bony mass. Although it seems to be stretched, the mucosa on top is still intact and has a normal color. Ulcerations may develop as a result of trauma or any mucosal lesion. They frequently start to appear throughout puberty and get larger over time. They usually do not self‐limit or pain.^36^

Radiographically, exostosis appears as a distinct round or oval calcified mass on top of the tooth roots. A biopsy should be performed if the diagnosis is unclear in any way. Tori and other exostoses share the same histologic traits. Hyperplastic bone is the term used to describe these growths, which are composed of mature trabecular and cortical bone.^3^ Individuals with multiple bony growths or lesions that are not in the typical locations for torus or buccal exostosis should be evaluated for Gardner syndrome. Intestinal polyposis and cutaneous cysts or fibromas are further signs of this autosomal dominant disorder.^38^, ^39^

### 1.2 Management

Usually, there is no need for treatment, but a surgical alveoloplasty may be necessary to remove the excess alveolar bone in those who may have periodontal problems,^18^ or if these bony protuberances cause pseudo‐swelling of the lip,^40^ masticatory dysfunctions,^18^ pain or discomfort for the patient, or disturbed smile esthetics.^41^, ^42^

The purpose of this paper is to present three orthodontically treated subjects with a history of TM and/or TP and formation of ABE during orthodontic treatment as well as to investigate the effect of biomechanical forces and the individual response on changes in alveolar bone thickness over time.

#### 1.2.1 Case I

**FIGURE 1:**
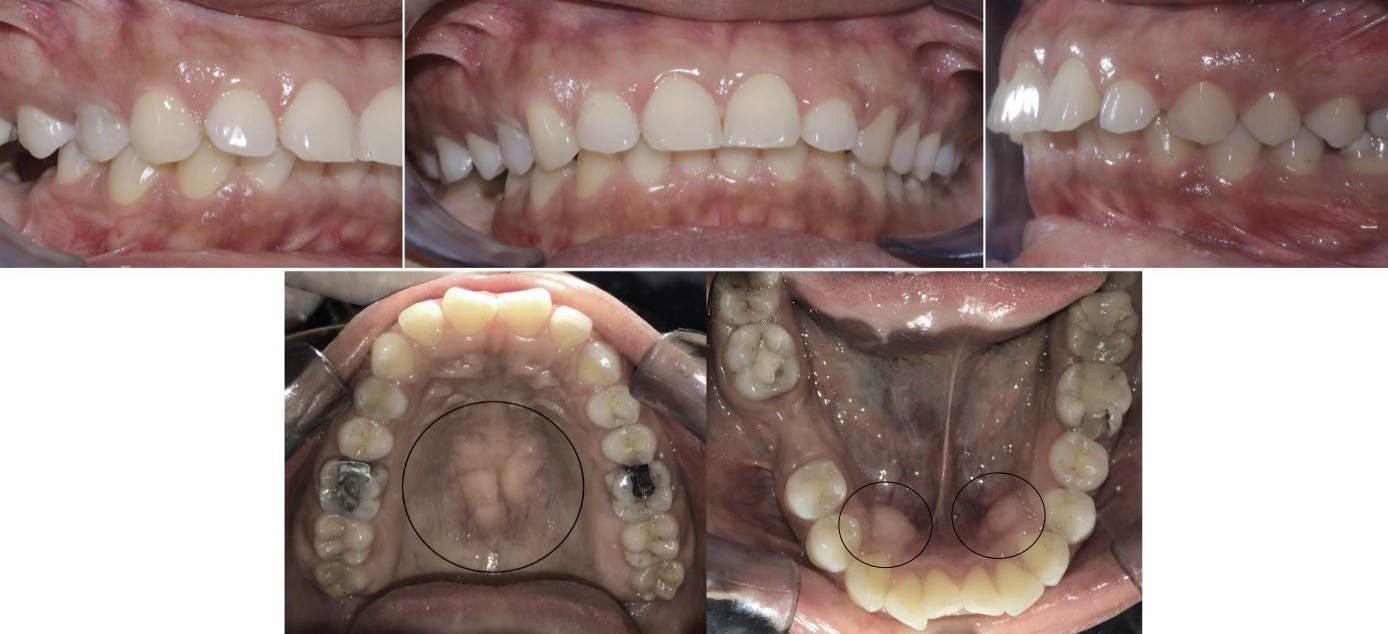
Intraoral pretreatment images showing the presence of TP and TM.

**FIGURE 2:**
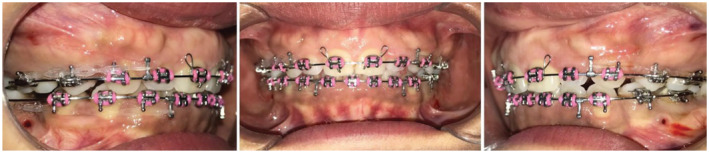
The appearance of ABE on the labial bone during retraction of upper anterior teeth.

**FIGURE 3:**
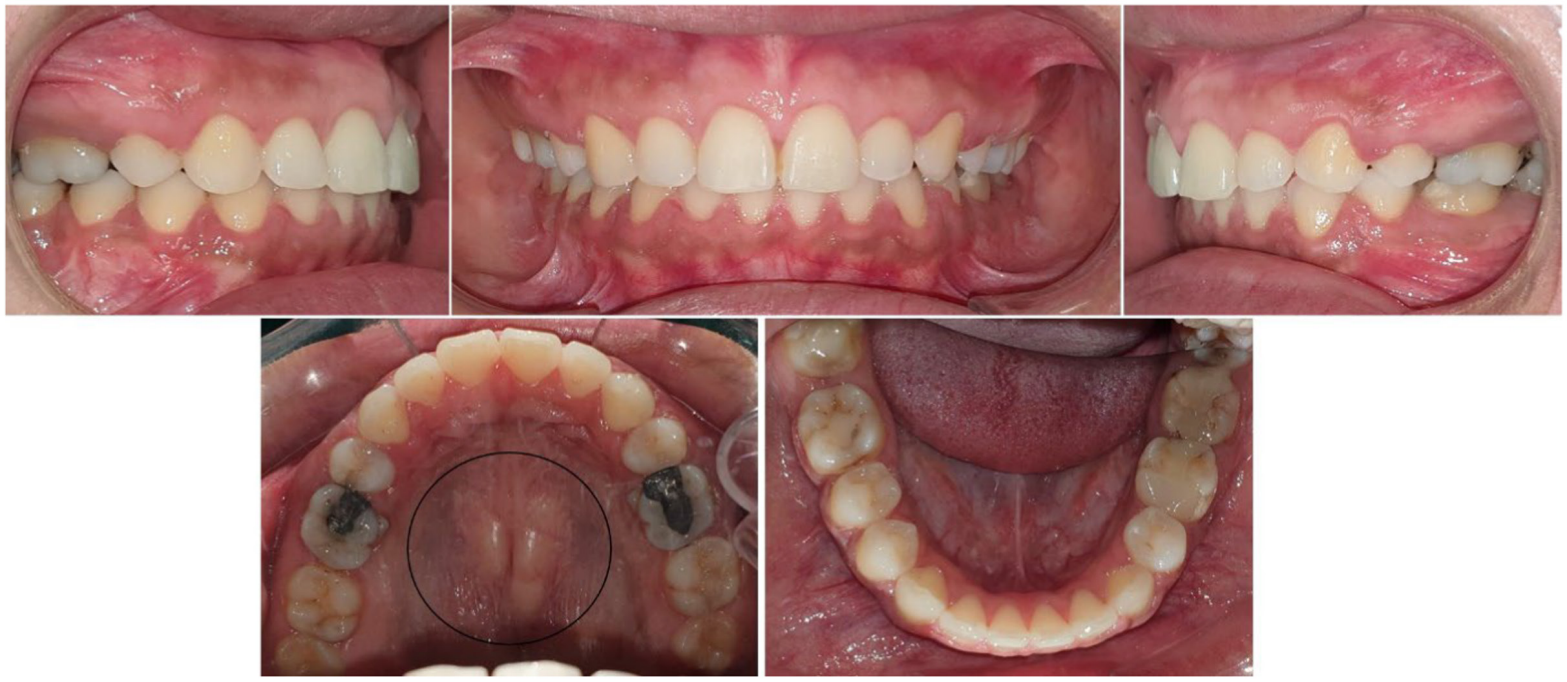
Regression of (ABE) spontaneously after 2 months of discontinuing orthodontic force at the end of treatment and subsequent debonding. Notice spontaneous regression of TM.

Nearly a year into her orthodontic treatment, a 30‐year‐old woman with a history of TP and TM (Figure 1) who had her upper premolars and lower left premolar extracted noticed bony outgrowths on the buccal mucosa during the space closure stages (Figure 2). A provisional diagnosis of ABE was obtained after a clinical examination, and it was agreed to carry on with regular orthodontic treatment. In order to see if spontaneous remission occurs, all orthodontic forces were stopped at the end of orthodontic therapy. The ABE spontaneously regressed after 2 months of force cessation (Figure 3). Once normal alveolar and gingival architecture was attained, the patient was debonded.

#### 1.2.2 Case II and Case III

**FIGURE 4:**
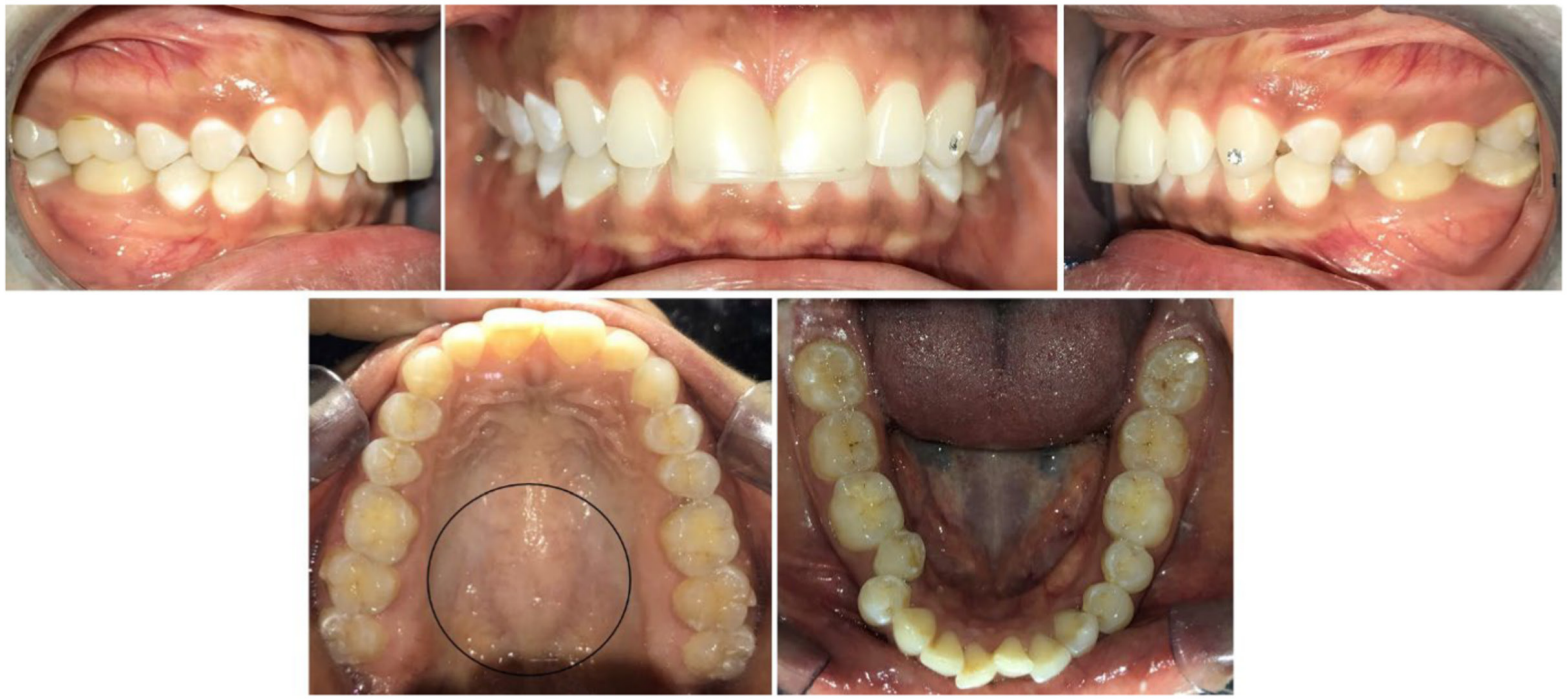
Pretreatment intraoral images showing presence of TP.

**FIGURE 5:**
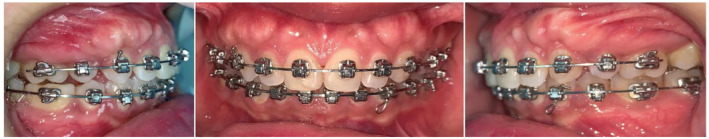
The presence of ABE in the upper arch even after cessation of the orthodontic forces at the end of orthodontic treatment.

**FIGURE 6:**
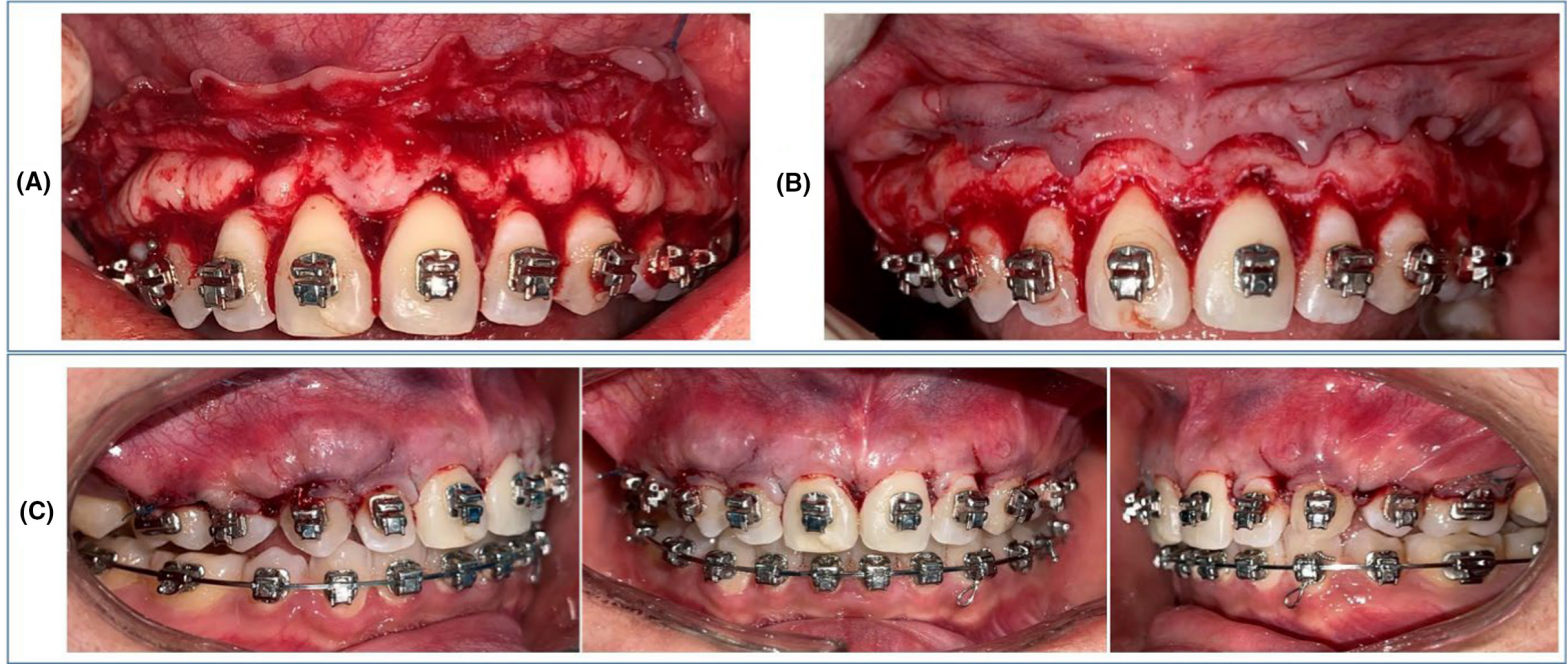
Surgical removal of ABE (A) sulcular incision was made and a full‐thickness mucoperiosteal flap was elevated; (B) Osteoplasty procedure using carbide bur and generous irrigation removing the bony nodules; (C) Sling sutures used to reposition the flap.

**FIGURE 7:**
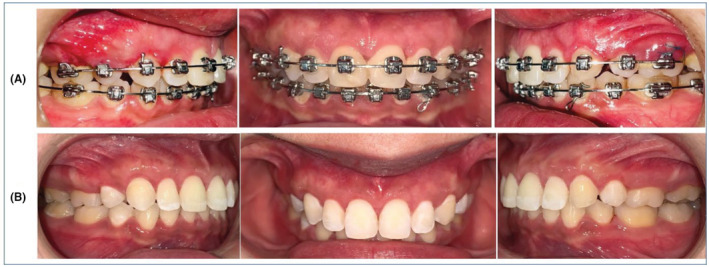
Postsurgical follow‐ups. (A) 11 days after surgery (B) 28 days after surgery.

**FIGURE 8:**
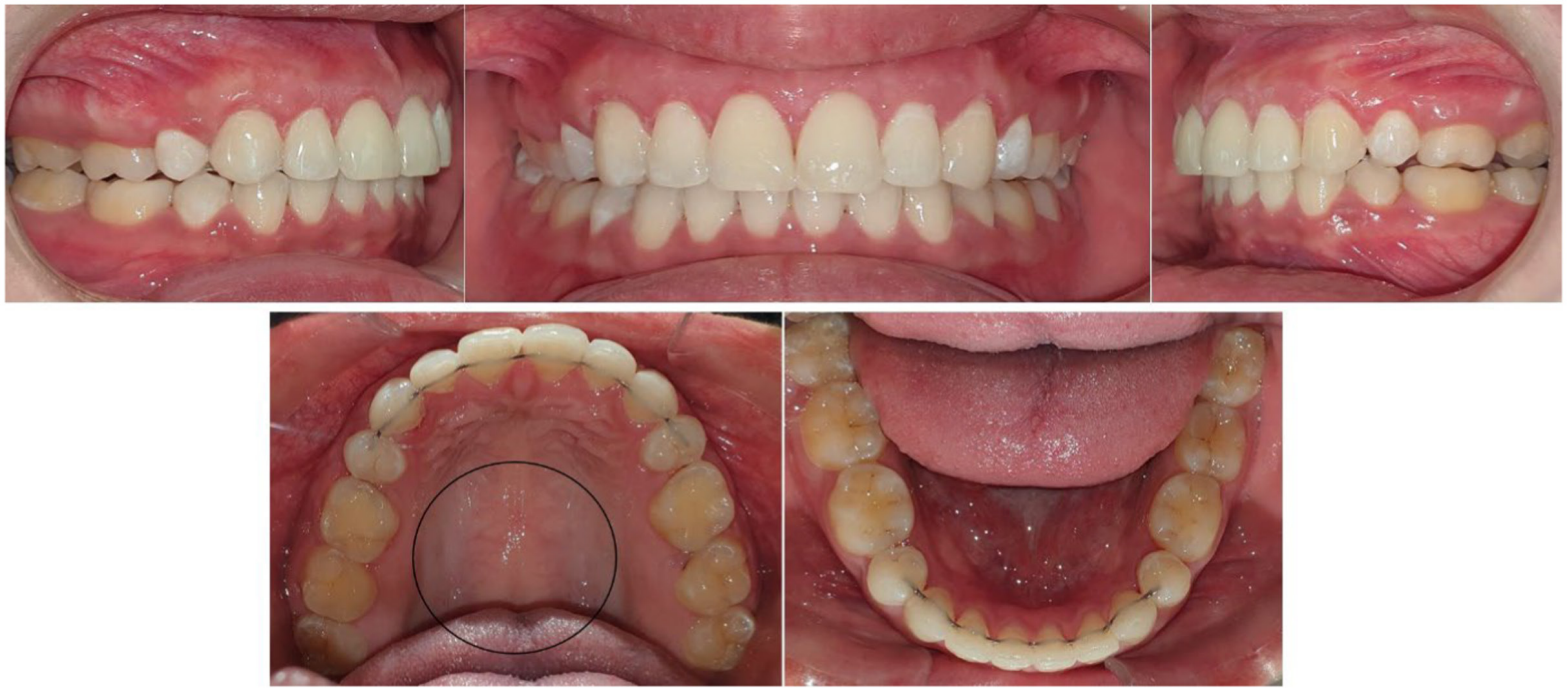
Post‐treatment intraoral pictures.

**FIGURE 9:**
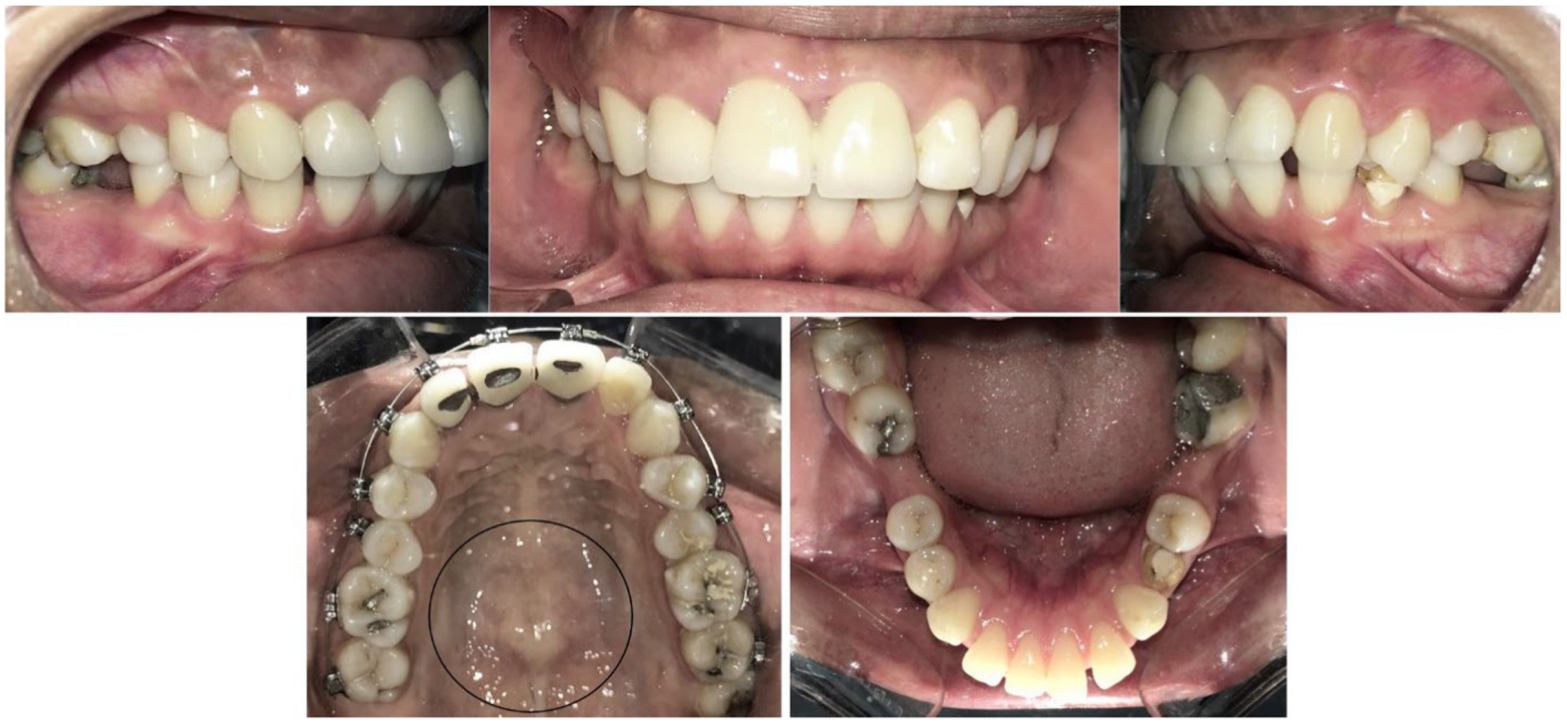
Pre‐treatment intraoral images.

**FIGURE 10:**
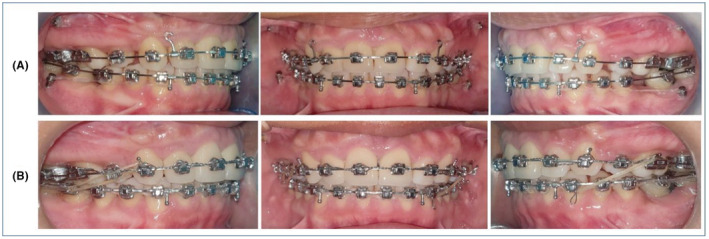
The development of ABE on the labial bone during upper anterior teeth retraction can be seen.

**FIGURE 11:**
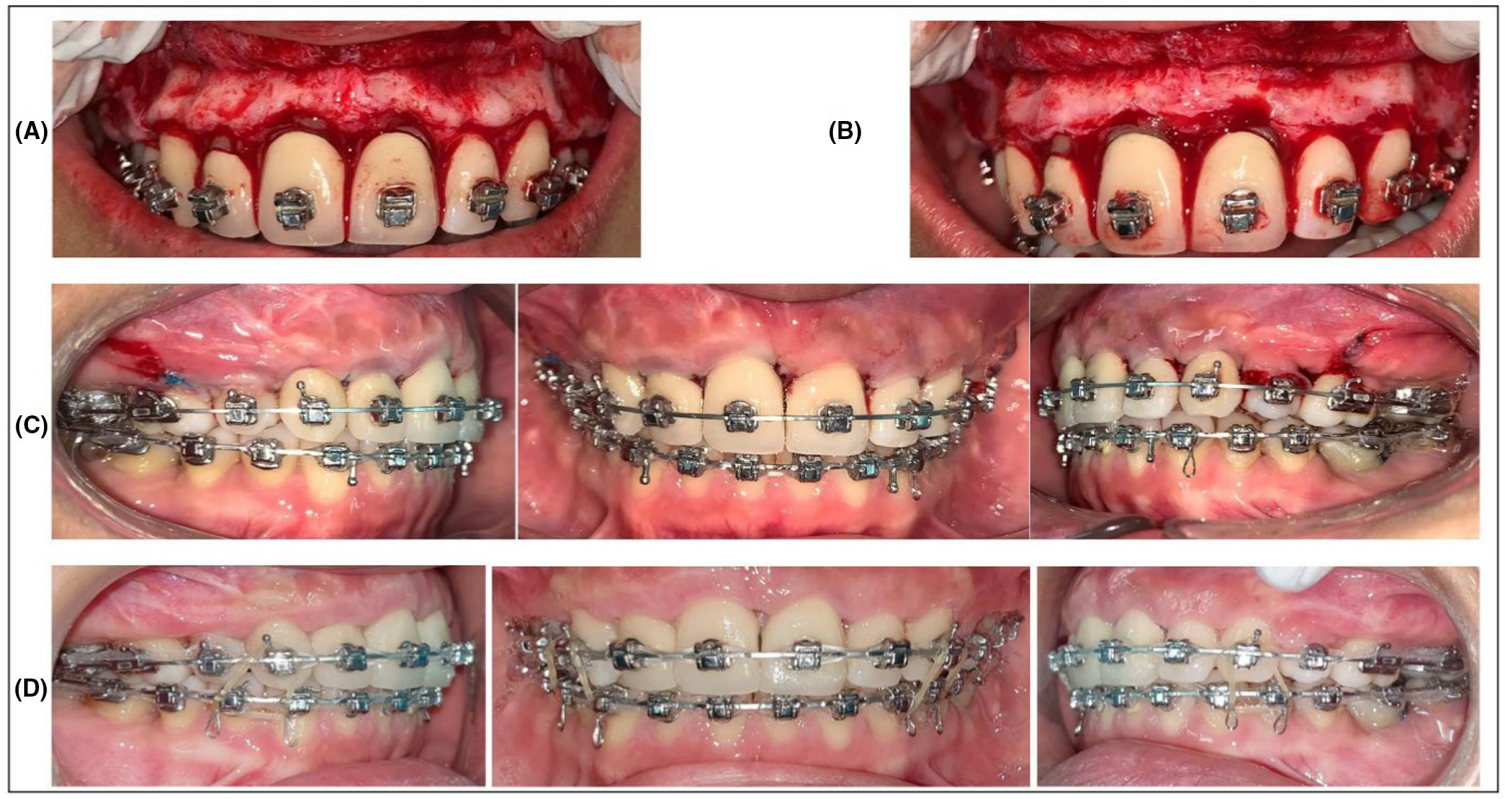
Surgical procedure and post‐operative images (A) sulcular incision was made and a full‐thickness mucoperiosteal flap was elevated; (B) Osteoplasty procedure completed and bony nodules were removed; (C) Sling sutures used to reposition the flap; (D) 11 days following surgery

**FIGURE 12:**
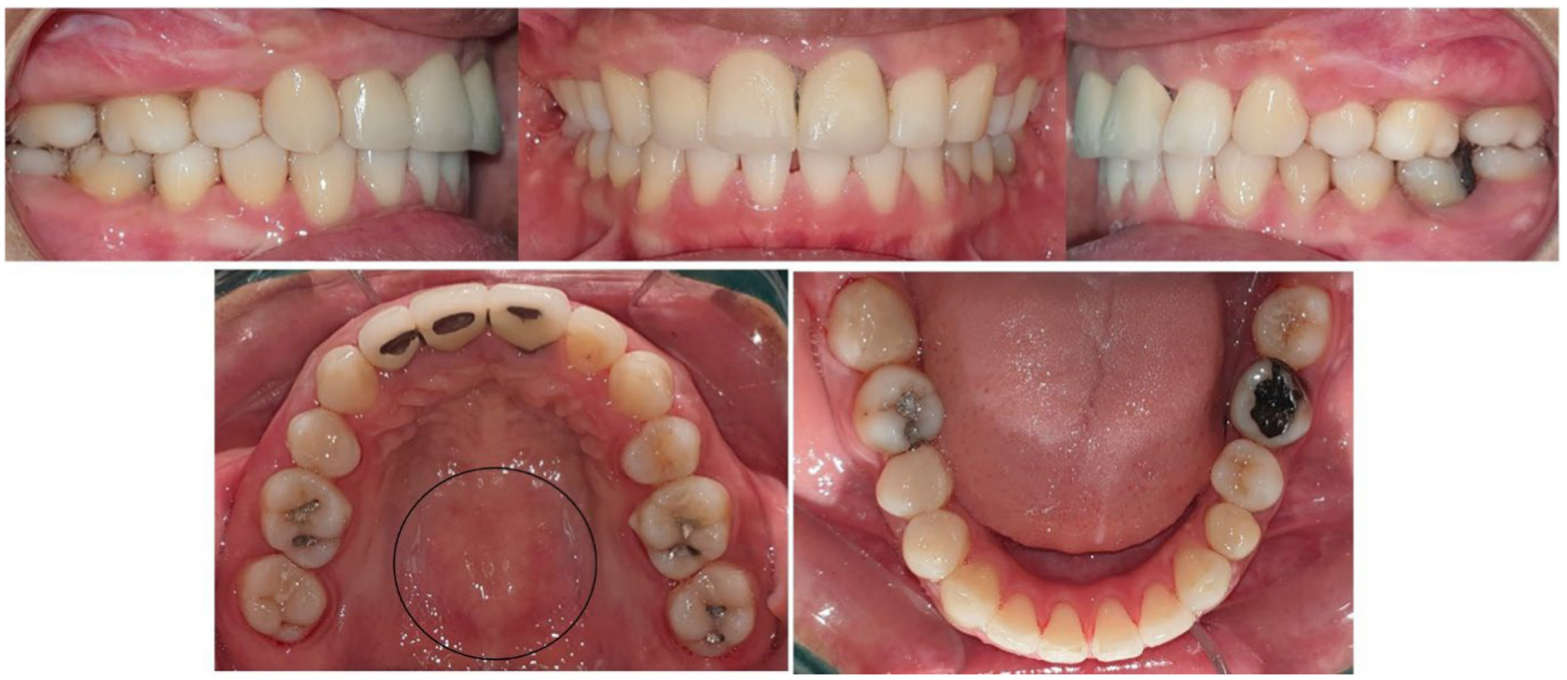
Post‐treatment intraoral images following removal of ABE.

In this section, we would like to discuss the diagnosis and subsequent treatment of two cases of ABE with a surgical intervention (Figures 4, 5, 6, 7, 8, 9, 10, 11, 12). We observed the establishment of ABE a year into orthodontic treatment in a 27‐year‐old female and a 28‐year‐old female with a history of palatal tori (Figures 4, 5, 9 and 10). To assess if the ABE had gone into spontaneous remission at the conclusion of the course of treatment, all forces were stopped. Following the cessation of forces for 3 months and still no change, the appropriate osseous surgery was scheduled for these patients. The patient's medical history was unremarkable, and the haemogram results were within normal ranges.

The patients' postoperative pain and recovery were assessed after 10 days and found to be uneventful. Braces were debonded and oral hygiene recommendations were emphasized. In the following month, follow‐up appointments to assess periodontal problems were scheduled.

### 1.3 Surgical procedure

The upper archwire was taken out, and then local anesthetic was administered. A sulcular incision was performed from the right first molar region to the left first molar region. When the full thickness mucoperiosteal envelop flap was elevated, the nodular osseous area could be observed. The bone growth was smoothed in line with all of the osteoplasty principles using a carbide bur and copious irrigation. A histopathological analysis of the bone that had been removed was performed. It had a thick trabecular bone that was consistent with the preliminary ABE diagnosis. Sling sutures were used to trim and reposition the flap (Figures 6 and 11). The patient also received postoperative instructions and medication with non‐steroidal anti‐inflammatory drugs (*NSAIDs*) and prophylactic antibiotics.

## 2 DISCUSSION

Similar to tori, there are several theories on the genesis of exostoses. The occlusal stress on the teeth in the afflicted areas may have been abnormally high or prolonged, which would explain the bony protrusion. The occurrence of tori has been found to be significantly correlated with tooth abrasion. While Reichart et al.^9^ noticed a significant correlation between tooth attrition and the presence of tori in the Thai population, they were unable to detect such an association in German people and advised against drawing any conclusion about the functional implications of this finding. Evidence of occlusal stress, such as that produced by clenching and grinding, was also shown to be significantly correlated with the existence of TM, as was reported by Kerdporn et al.^20^ Antoniades et al.^10^ proposed that the quasi‐continuous model of inheritance may also apply to buccal and palatal exostoses in light of their similarity in structure and position to TM. The authors concluded that the co‐occurrence of tori and exostoses in the same person is a highly exceptional event after documenting a third incidence of simultaneous TP with palatal and buccal exostoses.

However, prior to beginning orthodontic treatment, all three of the participants in this case series demonstrated a history of palatal tori. Jainkittivong et al.^43^ found that the co‐occurrence of TP and TM was also linked to a greater prevalence of exostoses and tori. 5.9% of their participants were found to have both buccal and palatal exostoses, it was also found. The results support Nery et al.'s^44^ theory that this group may represent a general type of multiple exostoses syndrome.

### 2.1 Orthodontics and ABE

Even though the precise process by which alveolar bone exostoses develop after orthodontic treatment is unknown, there is some evidence to support a link to the labial aspect of the alveolar bone thickening brought on by the rapid retraction of the upper incisors. The damage brought on by orthodontic forces, which results in the release of bone morphogenic proteins, which are expressed as exostoses and ossify at the stress points, may be the origin of the formation of buttressing bone. Rapidly retracted anterior teeth cause cortical bone remodeling to stall because it cannot keep up with the movement. It is a well‐known fact that cortical bone remodeling is influenced by the direction of tooth movement in the horizontal, vertical, and sagittal planes.^40^, ^45^, ^46^

There have also been reports of alveolar exostosis following the placement of orthodontic mini‐implants,^42^, ^47^ although the underlying reason was not identified. The cause may be excessive mechanical stress on the bone, which promotes the growth of osteogenic progenitor cells. Additionally, patients with tori or other bone exostoses are incredibly susceptible to ABE.^5^

Given this approach, it is logical to assume that tooth movement during orthodontic treatment may also be regarded as a microtrauma and may have a potential role in the emergence of oral exostoses.^40^, ^48^, ^49^ Yodthong et al.^41^ investigated how the thickness of their alveolar bone varied in response to the degree of intrusion, angulation, inclination, and rate of tooth movement. According to the study, when the incisors were retracted, the alveolar bone's thickness increased. Alveolar bone thickness fluctuations were significantly linked with tooth movement, inclination changes, and intrusion. The proportion of alveolar bone that is altered at the apical level depends on how much intrusion is performed when the upper incisors are retracted. Particularly, the alveolar crestal level labial bone thickness was adversely correlated with the upper incisors in the torque group and strongly positively correlated with the upper incisors in the tipping group.

A comprehensive systematic review^46^ also demonstrated that during en‐masse incisor retraction following extractions, alveolar bone thickness significantly increased on the labial side of the central incisors.

The complex etiopathogenesis of ABE may be traced back to the wide range of variables that affect the bone's capacity to remodel itself during retraction. The 2006 study by Tang et al.^50^ demonstrated that mechanical strain can generate morphological change and a magnitude‐dependent increase in the expression of bone morphogenic protein‐2, alkaline phosphatase, and collagen type I mRNA in osteoblast‐like cells, which may affect bone remodeling during orthodontic treatment.

We hope to conduct additional research in the near future to determine the correlation of ABE formation on orthodontically treated subjects with a history of TM or TP, as well as to investigate the effects of biomechanical force magnitude, force direction, force type (intermittent, continuous), extent of tooth movement, and individual response on changes in alveolar bone thickness over time in different skeletal malocclusions.^51^, ^52^

## 3 CONCLUSIONS

Our case study shows how alveolar bone exostoses form during orthodontic treatment. It is important to remember that each instance included a history of palatal tori. Our findings might be explained by the rate of incisor retraction and tooth movement, as well as a greater precedent of ABE formation in participants with preexisting palatal tori. Furthermore, we have successfully demonstrated surgical methods to eliminate ABE in the case that self‐remission does not take place after the cessation of orthodontic forces.

## AUTHOR CONTRIBUTIONS

**Adith Venugopal:** Conceptualization; investigation; methodology; writing – original draft; writing – review and editing. **Noem Bunthouen:** Data curation; formal analysis; investigation; methodology. **hasan sabah hasan:** Investigation; methodology; writing – original draft; writing – review and editing. **Krenare Agani:** Validation; visualization; writing – review and editing. **Andrea Butera:** Validation; visualization; writing – original draft; writing – review and editing. **Nikhilesh Vaid:** Project administration; validation; visualization; writing – review and editing.

## FINANCIAL INFORMATION

The authors received no financial support for the research.

## CONFLICT OF INTEREST STATEMENT

Authors declare no conflict of interests.

## DECLARATION OF PATIENT CONSENT

The authors certify that they have obtained all appropriate patient consent.

## CONSENT

Written informed consent was obtained from the patient to publish this report in accordance with the journal's patient consent policy “on the title page of the manuscript.”

## Data Availability

The data that support the findings of this study are available from the corresponding author upon reasonable request.
